# Comment on “Simulating Radiotherapy Effect in High-Grade Glioma by Using Diffusive Modeling and Brain Atlases”

**DOI:** 10.1155/2015/801057

**Published:** 2015-11-22

**Authors:** Giovanni Borasi, Alan E. Nahum

**Affiliations:** ^1^The Italian National Research Council (CNR), Institute of Bioimaging and Molecular Physiology (IBFM), Edificio LITA, Via F.lli Cervi 93, Segrate, 20090 Milan, Italy; ^2^Clatterbridge Cancer Centre, Bebington CH63 4JY, UK

The paper by Roniotis et al. [1] contains a serious error. The problematic term is *f*(*c*) representing the effect of radiotherapy on the tumor (equation (11) page 4). This term is inserted in the reaction-diffusion (Fisher-Kolmogorov) equation (3), describing the expansion-infiltration of brain tumors. As is well known, in a mathematical expression, adding (or subtracting) two terms with different dimensions is invalid (e.g., what would be the result obtained by adding a length with an area?). That is exactly what has been done in equation (11), first line, that we rewrite here, adding square brackets for greater clarity: 
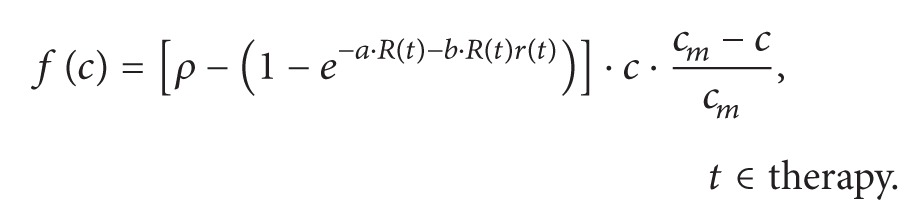
(11)The term included in the curved brackets is a simple number (i.e., dimensionless), but *ρ* has the dimensions of 1/time and represents the tumor expansion rate. Therefore one cannot subtract the (1 – exponential) term from *ρ*. It is probable that all the subsequent results are erroneous (in particular Figure 2).

The “dimensional” control of an equation is so important in physics that it is included in the program of undergraduate courses. In the present case, the error may have serious consequences in the context of applications of physics to medicine, particularly, regarding the treatment of brain tumors.

## References

[1] Roniotis A., Marias K., Sakkalis V., Manikis G. C., Zervakis M. (2012). Simulating radiotherapy effect in high-grade glioma by using diffusive modeling and brain atlases. *Journal of Biomedicine and Biotechnology*.

